# ﻿*Paracladopuschiangmaiensis* (Podostemaceae), a new generic record for China and its complete plastid genome

**DOI:** 10.3897/phytokeys.195.82789

**Published:** 2022-04-20

**Authors:** Mingsong Wu, Kai Zhang, Xinquan Yang, Xin Qian, Rongtao Li, Jianhe Wei

**Affiliations:** 1 Hainan Provincial Key Laboratory of Resources Conservation and Development of Southern Medicine, Hainan Branch of the Institute of Medicinal Plant Development, Chinese Academy of Medical Sciences & Peking Union Medical College, Haikou 570311, Hainan, China Chinese Academy of Medical Sciences & Peking Union Medical College Haikou China; 2 Ministry of Education Key Laboratory for Ecology of Tropical Islands, College of Life Sciences, Hainan Normal University, Haikou 571158, Hainan, China Hainan Normal University Haikou China; 3 College of Life Sciences, Fujian Agriculture and Forestry University, Fuzhou 350002, Fujian, China Fujian Agriculture and Forestry University Fuzhou China; 4 Institute of Medicinal Plant Development, Chinese Academy of Medical Sciences & Peking Union Medical College, Beijing 100193, China Chinese Academy of Medical Sciences & Peking Union Medical College Beijing China

**Keywords:** aquatic, chloroplast genome, *mat*K, new generic record, *Paracladopus*, river-weeds, Wuzhi Mountain

## Abstract

The genus *Paracladopus* was established based on the type species *P.chiangmaiensis* in 2006. The two *Paracladopus* species are distributed in Thailand and Laos; however, neither of them has been documented in China to date. During our field work in 2020, we collected a river-weed in Wuzhi Mountain, Hainan Province of China. After checking the morphological characters, it was identified as *P.chiangmaiensis*. Then, we assembled and annotated its chloroplast genome based on the genome skimming data. The results showed that the complete chloroplast genome was 133,748 bp with 35% GC content, consisting of 76 protein-coding genes, 30 tRNA genes, and 4 rRNA genes. A maximum-likelihood tree constructed based on the *mat*k genes showed that WuMS109 was clustered with *P.chiangmaiensis* (AB537420, AB698348) without base difference and together with the remains of *Paracladopus* formed a sister clade to *Cladopus*. This is the first report of *P.chiangmaiensis* that represents a new generic record for China. The discovery of this river-weed could lay the foundation for investigating their biogeographical patterns and species evolution in further studies.

## ﻿Introduction

Podostemaceae, also known as river-weeds or podostems (Gustafsson et al. 2002; Koi and Kato 2012), which are the largest family of strictly aquatic angiosperms (De Mello et al. 2011), grow attached to waterworn rock surfaces, stones or even wood in rapids and waterfalls with pristine hydrology and high water quality (Rutishauser 1995; Koi and Kato 2020; Xue et al. 2020). They are found worldwide in rivers or streams with open and sunny habitats (Werukamkul et al. 2016), but distributed mainly in the tropical to warm-temperate regions with seasonal climates (Kato 2011). The species diversity center of river-weeds is located in the equatorial region of South America (De Mello et al. 2011). Its habitat and morphology have obviously undergone high specializations and extensive reductions (Gustafsson et al. 2002). These species lack double fertilization and the vascular tissue is reduced or lost (Johri et al. 1992; Kato 2016; Khanduri et al. 2016). The vegetative body of most river-weeds resembles algae, lichens, or mosses. So, they are easily overlooked and more field works are needed to investigate the species diversity. River-weeds are usually not obvious at conventional demarcation of stem, leaf and root (Willis 1902). The interpretation of the vegetative body also evokes much controversy (Mohan Ram and Sehgal 1992; Ota et al. 2001; Sehgal et al. 2002). In this text, we adopt the classical root-shoot model with its structural categories ‘roots’, ‘shoots’. The term ‘root’ refers to photosynthetic structures when endogenous shoot buds are developed but there are no exogenous leaves. Shoots are apparently absent or reduced, borne adventitiously on root. The Podostemaceae show an amazing diversity of root types. They vary from thread-like to ribbon-like and further to crustose (Mathew and Satheesh 1997; Jäger-Zürn 2000; Koi et al. 2019; Krishnan et al. 2019).

The life cycle of river-weeds is dictated by high and low water periods (Lalruatsanga 2020). During the rainy season, the plants submerge in violent currents exclusively in the vegetative phase (Kato 2016). When the water level lowers during the dry season, the plants emerge from the water and enter into the reproductive phase (Kelly et al. 2010; Koi and Kato 2012, 2015). In the early dry season, the plants form flower buds underwater, and subsequently come into flower and fruit when exposed to the air. The exposed plants wither and die while seeds are dispersed from their capsules. They are annual herbs, but perennial when submerged all year round (Kato et al. 2019). Exposure is necessary for reproduction, otherwise the plants will be unable to complete their life cycle (Kato 2016).

Podostemaceae comprises ca. 351 species (excluding infraspecies) assigned to ca. 50 genera in three subfamilies (POWO 2021). Podostemoideae, the largest subfamily (ca. 43 genera and ca. 322 species) of Podostemaceae, is distributed worldwide; subfamily Tristichoideae (6 genera and ca. 28 species) is mainly distributed in Asia, with the exception of *Tristichatrifaria* (Bory ex Willd.) Spreng., which is widely dispersed from Central and South America to Africa; while subfamily Weddellinoideae (a monotypic genus) is confined to South America (Kato 2006a). The phylogenetic position of Podostemaceae has been revealed by molecular data. They were included in the order Malpighiales, forming a sister group with Hypericaceae and related to Clusiaceae and Callophyllaceae based on plastid and nuclear markers or plastoms (APG IV 2016; Magallón et al. 2015; Li et al. 2021). However, Podostemaceae was a more basal isolated-clade in rosids and was recovered as Podestemales according to nuclear genes (Baker et al. 2021) or genomes (Xue et al. 2020).

The genus *Paracladopus* M.Kato, belonging to subfamily Podostemoideae, was established by Kato in 2006 based on the type species *P.chiangmaiensis* M.Kato, which was first discovered in northern Thailand (Kato 2006b). Two years later, another new species *P.chantaburiensis* Koi & M.Kato was discovered in southeastern Thailand (Koi et al. 2008). The two *Paracladopus* species are distributed in Thailand and Laos; however, neither of them has been documented in China to date. The ribbon-like roots and ellipsoidal or globose capsule of *Paracladopus* are similar to *Cladopus* H.Möller, but can be distinguished by its capsules with ribs or stripes, shoots borne on the sinuses of root branching and flanks of the root between successive root branches, and holdfasts presenting on ventral surface of root under the shoots (Koi and Kato 2012, 2015). Molecular phylogenetic analysis showed that *Paracladopus* was sister to *Cladopus*, and the two genera were in turn sister to the ‘*Hydrobryum*’ clade (including *Ctenobryum* Koi & M.Kato, *Hydrodiscus* Koi & M.Kato, *Hanseniella* C.Cusset, *Hydrobryum* Endl. and *Thawatchaia* M.Kato, Koi & Y.Kita) (Koi et al. 2012, 2019).

**Figure 1.**
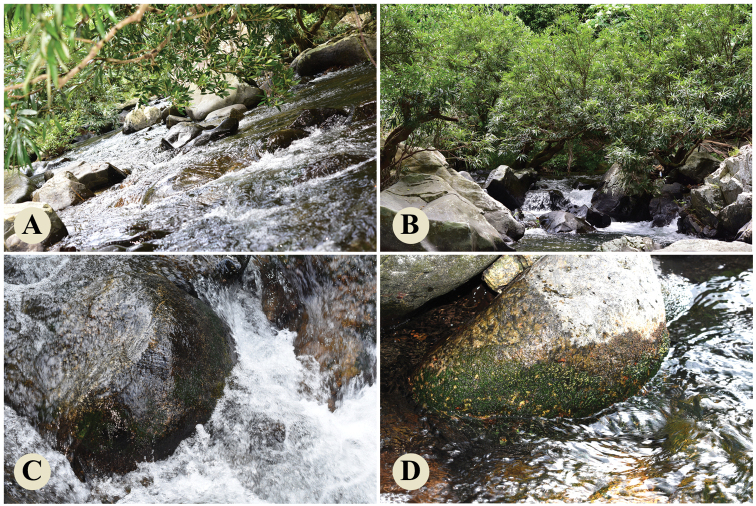
The natural habitat of *Paracladopuschiangmaiensis* in Wuzhi Mountain **A, B** the river-weed submerging in water with open and sunny habitat **C** the river-weed attached to waterworn rock surfaces in rapids **D** several individuals emerging from water and enter into reproductive phase.

**Figure 2.**
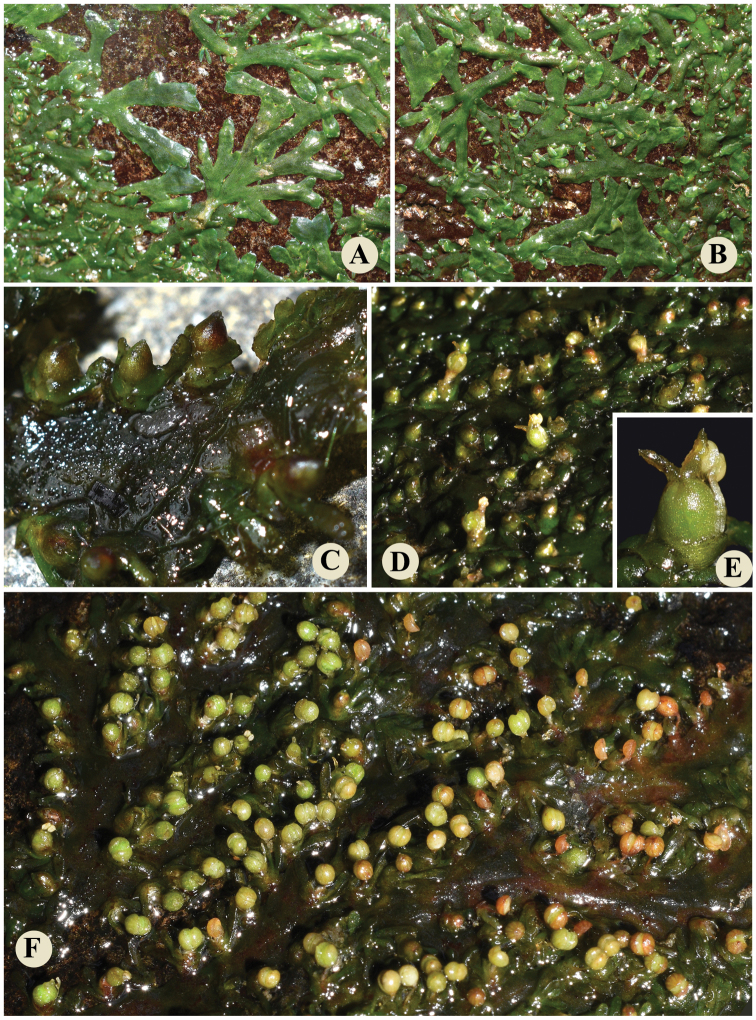
*Paracladopuschiangmaiensis* in natural habit **A** inconspicuous shoots are observed **B** ribbon-like roots with tufts of leaves on flanks **C** flowering shoots on flanks of root **D, E** flowers with indehiscent anthers **F** nearly ripe fruits with dehiscent capsule valves.

During a field trip to Wuzhi Mountain, Wuzhishan City, Hainan Province, China in December 2020, a river-weed (Fig. 1A-C) without flower and fruit attached to rock surfaces in rapids caught the first author’s attention. At first sight, it resembled *Cladopus* species by its ribbon-like structure; however, ensiform leaves and shoots borne on both sides of the ribbon-like structure (Fig. 2A, B) confused us. In March 2021, we revisited Wuzhi Mountain and found a few residual flowers and a large number of mature fruits. The flower possessed a single stamen and a single pistil with 2 stigmas (Fig. 2D, E). The fruit was globose with some stripes. After a survey of literature, we confirmed the morphological characters of the plants matched that of the species *Paracladopuschiangmaiensis*, distributed in Thailand and Laos. This species and genus has not been previously reported to occur in China. So, its discovery in Hainan Province represented the first record of this genus for China.

## ﻿Materials and methods

### ﻿Material

Plant samples were collected from Wuzhishan Tropical Rainforest Scenic Area in Shuiman Village, Hainan Province (18°52'2.68"N, 109°40'51.41"E). The voucher specimen (Mingsong Wu, WuMS109) was deposited in the Traditional Chinese Medicine Herbarium of Hainan Province.

### ﻿Total DNA extract, genome assembly and annotation

Total genomic DNA was extracted from the entire thallus dried immediately by silica gel using a modified CTAB method (Doyle and Doyle 1987). Genome skimming was performed using next-generation sequencing technologies on the Illumina Novaseq 6000 platform with 150 bp paired-end reads and 350 bp insert size by Novogene Bioinformatics Technology Co. Ltd. (Tianjin, China). A total of 3.85 Gb paired-end sequencing data was generated to assemble plastome using GetOrganelle pipeline (Jin et al. 2020b). The assembly graph viewer Bandage (Wick et al. 2015) was used to visualize the assemblies. The program Plastid Genome Annotator (Qu et al. 2019) was employed as the annotation tool with *Tristichatrifaria* (NC_049109) designated as the reference. Geneious Prime 2020.1.2 (Biomatters Ltd., Auckland, New Zealand) was used to adjust the start/stop codons, intron boundaries and tRNA genes for the preliminary annotation result. The *mat*K gene was extracted from the chloroplast genome.

### ﻿Phylogenetic analyses

To confirm our identification results based on morphological characters, and reveal the phylogenetic relationship of this species within *Paracladopus* and closely related genera, we downloaded the *mat*k gene sequences from Genbank as shown in Fig. 6, which contains 24 species from 8 genera. Among them, three species of *Podostemum* were used as outgroups. Based on the GTRGAMMA substitution model and 1000 bootstraps, we constructed a maximum likelihood tree using the RAxML-HPC2 (Stamatakis 2014) on XSEDE (8.2.6) in Cipres Science Gateway (Miller et al. 2012).

## ﻿Results and discussion

### ﻿New generic record for China

We discovered *Paracladopuschiangmaiensis* from Hainan province, representing a new generic record for China. Currently, only one population was discovered in Wuzhishan Tropical Rainforest Scenic Area. They live in turbulent rivers adhering to submerged rock surface with open and sunny habitat as shown in Fig. 1. When the water level dropped in the dry season, the plants were exposed to air to produce flowers and fruits shortly afterwards (Fig. 1D).

**Figure 3.**
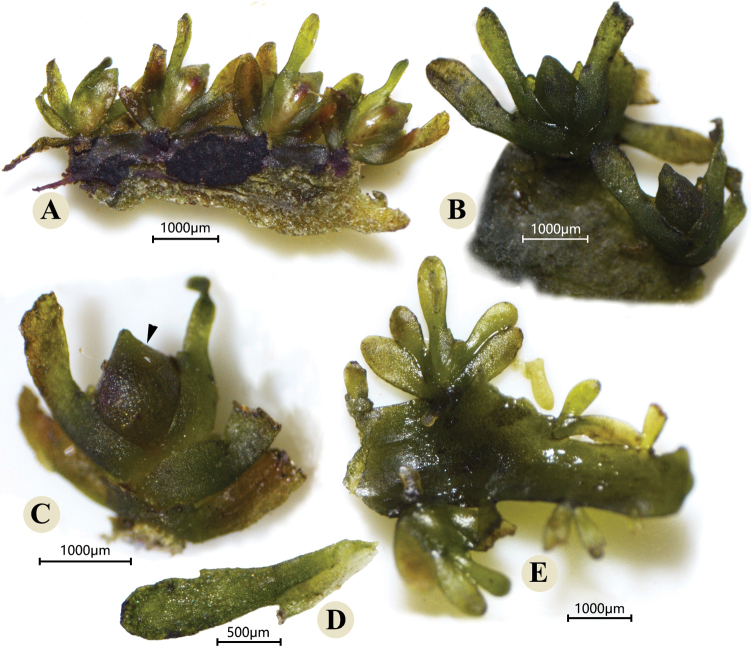
Morphology of *Paracladopuschiangmaiensis* shoots **A** four flowering shoots bone on the root **B** reproductive shoot with ensiform bracts and terminal floral bud enclosed by spathella **C** lateral view of ensiform bracts covering flower bud, arrow means spathella **D** ensiform leaves with sheaths on the inner edge **E** vegetative shoots bone on flanks of root.

**Figure 4.**
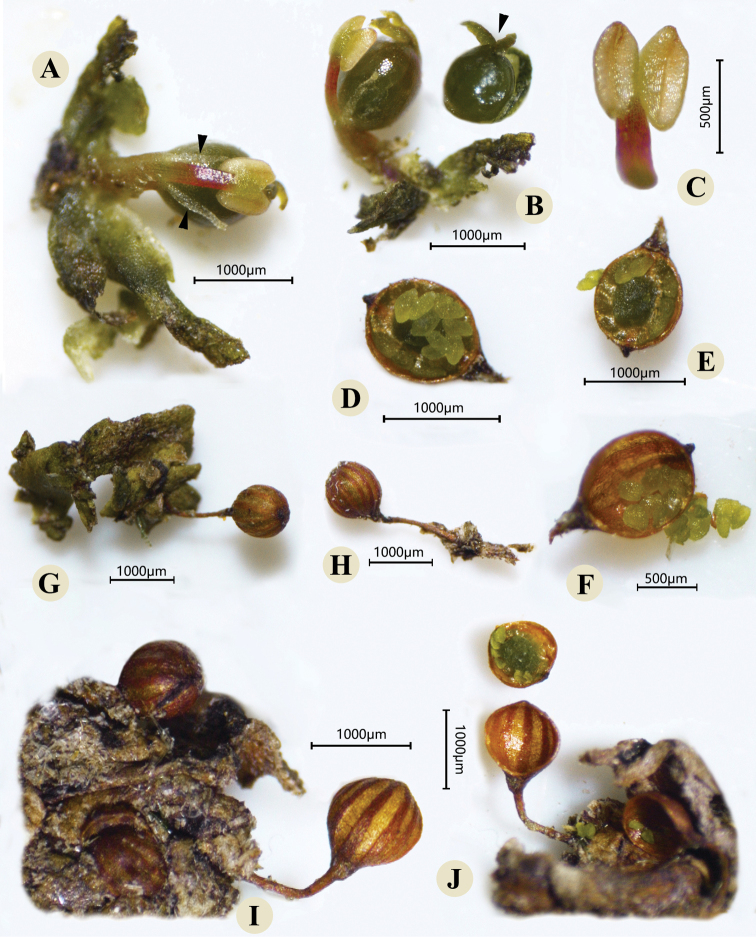
Flower and fruit of *Paracladopuschiangmaiensis***A** a flower bud (spathella removed), arrows mean two tepals on sides of stamen **B** lateral view of a flower bud (spathella and bracts removed), showing single stamen and two stigmas (as shown by the arrow) **C** stamen with indehiscent anther **D, E, F** fruit and seeds **G, H** stalked capsules covered with stripes **I, J** two persistent capsule valves with some stripes.

The roots, shoots, flowers and capsules of *P.chiangmaiensis* in the natural habitat were recorded in Fig. 2. Meanwhile, photographs under the stereoscope based on the fixed material were taken and shown in Figs 3, 4. The shoots were borne on the flanks of root and the sinuses of root branching (Figs 2B, 3A, E). The flowering shoots were very short with 4–6 ensiform bracts (Figs 2C, 3B, C). During the flower bud period, the pistil and stamen were covered by a spathella (Figs 2C, 3C). Flower was terminal in the short-shoot composed of one stamen with one linear tepal on each side (Fig. 4A) and one pistil with two stigma and a smooth ovary (Figs 2D, E, 4B). Capsule stalked was globose with 10–14 narrow stripes (Figs 2F, 4G, H), and dehisced by two equal valves (Fig. 4I, J). The flowering and fruiting period is from January to March. All morphological characteristics of this river-weed newly discovered in Wuzhi Mountain in China are consistent with the species described by Kato in Thailand and Laos (Kato 2006b; Koi and Kato 2012). The discovery of this river-weed represents a new generic record for China, and lays the foundation for investigating their biogeographical patterns and species evolution in further studies.

### ﻿The complete plastid genome

**Figure 5.**
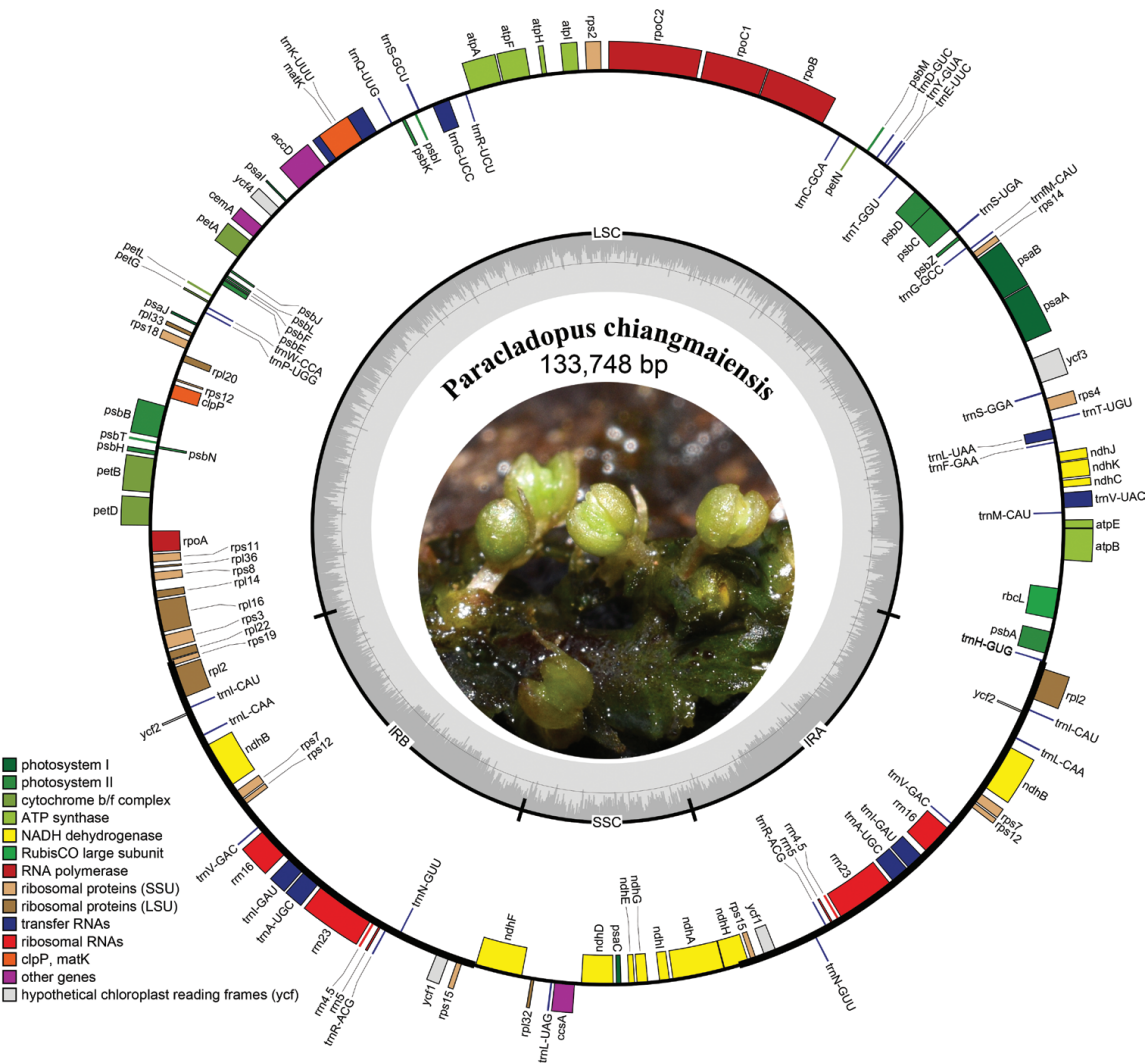
Gene map of *Paracladopuschiangmaiensis* complete chloroplast genomes. Genes inside the circle are transcribed clockwise, genes outside the circle counter clockwise. The circle inside the GC content graph marks the 50% threshold.

The Podostemaceae possess one of the smallest known plastid genomes among the Malpighiales covering approximately 130 kb in length (Bedoya et al. 2019; Jin et al. 2020a). The complete chloroplast genome of *P.chiangmaiensis* was successfully assembled and annotated. The result showed that the length of the complete chloroplast genome was 133,748 bp with 35% GC content, consisting of a typical quadripartite plastid structure with two inverted repeats (20,854 bp) separated by a large single-copy region (79,537 bp) and a small single-copy region (12,503 bp) (Fig. 5). *Paracladopuschiangmaiensis* contained 110 unique genes, including 76 protein-coding genes, 30 tRNA genes, and 4 rRNA genes, and lost *rps*16 gene, *rpl*23 genes, the intron of the *clp*P gene and *rps*12 gene, and the second intron of the *ycf*3 gene. An uncommon loss of *ycf*1 and *ycf*2 and a major inversion over 50 kb were also found. The inversion contained 46 genes spanning from *trn*K-UUU to *rbc*L in the large single-copy region. The *rps*15 gene relocated from SSC to IR for the expansion of IRs. GC content in the IRs was higher than in other regions of the plastid. All plastid characters of *P.chiangmaiensis* were similar to other plastome reported in Podostemaceae, excluding *Tristichatrifaria* whose *rps*15 gene was located at the SSC/IRA boundary (Bedoya et al. 2019; Jin et al. 2020a). The annotated plastome was deposited in GenBank under the accession number MZ645928. Our results could provide essential data to investigate the phylogeny and evolution of river-weeds in the future.

### ﻿The phylogenetic analysis

**Figure 6.**
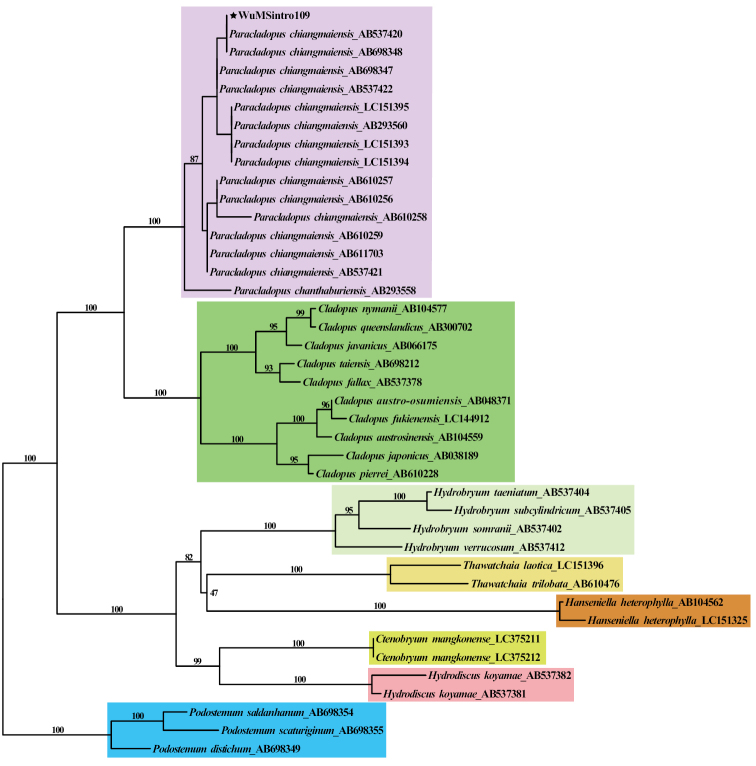
The plastid phylogeny of the *Paracladopus* and closely related genera. Maximum-likelihood (ML) tree inferred from *mat*K gene. The number at each node indicates the ML bootstrap values. Species are color-coded according to genus. Three species of *Podostemum* (sky blue) are designed as outgroups. The sequence newly generated in this study marked by black pentagram.

The *mat*K gene sequence extracted from complete chloroplast genome of Wuzhishan river-weed (WuMS109) was 1,527 bp in length. The phylogenetic relationship constructed based on the *mat*K gene sequences showed that WuMS109 and *P.chiangmaiensis* (AB537420, AB698348) were clustered together without base difference (Fig. 6), which was consistent with the result of morphological identification, so, the river-weed in Wuzhi Mountain was identified as *P.chiangmaiensis* with certainty. The genus *Paracladopus* is similar to *Cladopus* in ribbon-like roots and globose or ellipsoidal capsule, but can be distinguished by its shoots borne on the sinuses of root branching and flanks of the root, holdfasts presenting on ventral surface of root under the shoots and capsules with ribs or stripes. According to the key in ‘Flora of China’ (Qiu and Philbrick 2003), the pericarp of capsule with ribs denoted the genus *Hydrobryum* in Podostemaceae, but roots crustose and tufts of linear leaves scattered on its dorsal surface made the *Hydrobryum* markedly distinct from *Cladopus*. Molecular phylogenetic analysis showed that *Paracladopus* was a sister to *Cladopus*, but distantly related to *Hydrobryum*. The phylogenetic result was consistent with the previous research (Koi et al. 2012; Kato et al. 2019).
